# Exploring the Relationship Between Organizational Culture Types and Knowledge Management Processes: A Meta-Analytic Path Analysis

**DOI:** 10.3389/fpsyg.2022.856234

**Published:** 2022-05-30

**Authors:** Riad Aichouche, Khalil Chergui, Said Khalfa Mokhtar Brika, Mohammed El Mezher, Adam Musa, Ahmed Laamari

**Affiliations:** ^1^Department of Management Sciences, Faculty of Economics, Commercial and Management Sciences, COFIFAS Laboratory, University of Oum El Bouaghi, Oum El Bouaghi, Algeria; ^2^Department of Administrative Sciences, Applied College, University of Bisha, Bisha, Saudi Arabia; ^3^Faculty of Science and Arts, University of Bisha, Bisha, Saudi Arabia

**Keywords:** organizational culture, Competing Values Framework, knowledge management processes, meta-analytic path-analysis, creation, dissemination, storage, application

## Abstract

This study investigated the relationship between organizational culture types according to Competing Values Framework (Clan, Adhocracy, Market, Hierarchy) and Knowledge Management Processes (Creation, Dissemination, Storage, Application) using meta-analytic path analysis. To produce the necessary pooled correlation matrix for model testing, we used the univariate (r) approach to carry out two additional meta-analyzes. Based on data collected from several research databases, we extracted the paired correlation coefficients (r) among knowledge management processes (*k* = 32, *N* = 6835) then the inter-correlations between knowledge management processes and culture types (*k* = 7, *N* = 865). The findings revealed that no particular culture type has a stronger effect on all KM processes. Clan, Adhocracy and Market have significant but varying effects on Knowledge Management processes. Notably, the clan is more associated with knowledge creation, while Adhocracy has a greater effect on knowledge application, and market has a stronger effect on knowledge dissemination and storage. However, hierarchical culture has an insignificant effect on knowledge creation and the lowest effects on the rest of Knowledge Management processes. Therefore, the study concluded that knowledge management success is determined by developing a balanced portfolio of cultural traits from clan, adhocracy and market cultures.

## Introduction

Every organization must adopt new management tools to compete and survive in the current changing business environment. In today’s knowledge economy, organizations are knowledge and learning systems, and the way they are learning and managing knowledge is reflected in the way they are organized (Taylor and Oinas, 2006; Alam, 2019). Gottschalk (2005) argued that the knowledge-based view of the firm suggests that organizational success depends on heterogeneous knowledge flows and making suitable knowledge integration mechanisms to enhance market response capabilities. This heterogeneity needs to be maintained over time because it explains why an organization is different (Donate and Guadamillas, 2010; Cepeda-Carrion et al., 2017). Therefore, knowledge management (KM) is commonly regarded as a critical requirement for obtaining a competitive advantage over competitors. However, organizations are still struggling to find an effective way to successfully managing their knowledge assets.

Recognizing that organizational culture (OC) plays a significant role in KM success increases the enormity of the task. Although numerous studies have addressed the impact of cultural factors on KM practices, there are no widely generalizable results (Jacks et al., 2012). In the literature, some scholars addressed the association between certain cultural factors and one or more KM processes. Jacks et al. (2012), for example, identified a range of culture dimensions such as trust, control orientation, power, obligation, and openness in their meta-analysis study. However, others like (Devi et al., 2007) were more interested in studying the effects of culture types according to the Competing Values Framework (CVF), as this offers a practical assessment of OC and a valuable tool to analyze OC in relationship to other variables. Thus, such adoption could lead to a better understanding of the OC-KM relationship.

Concerning KM processes, some studies were interested in addressing the effect between OC types and a single KM process, particularly knowledge sharing (Suppiah and Singh, 2011; Andam, 2017; Memon et al., 2020) or with several KM processes (Kangas, 2009; Chidambaranathan and Swarooprani, 2017). Nevertheless, the results of these studies are still controversial. There is no consensus regarding which culture type is appropriate for KM practices.

The current study compiles prior research aiming to examine and understand how OC types affect KM processes. In this regard, this paper used meta-analytic structural equation modeling (MASEM) to elucidate the impact of OC types, namely Clan, Adhocracy, Market, and Hierarchy on KM processes (creation, storage, dissemination, and application).

The remainder of the article is structured as follows: we briefly review the existing topic literature. Then, we present the steps of the meta-analytic path model method. The following section provides the results of the empirical study of the relationship between OC and KM processes and the path model fitting results. The last section discusses the results and sets out the limitations and future lines of research.

## Literature Review

### Organizational Culture Types

Organizational Culture is one of the most significant subjects in organizational behavior which has been studied and defined variously (Ahmady et al., 2016). OC is typically defined as a set of assumptions, symbols, organizational beliefs, routines, shared language, and myths; it appears in the way people behave and making sense (Lee J.-C. et al., 2016; Alam, 2019). Additionally, OC is intrinsically linked to all facets of an organization’s functioning, making it an ambiguous and difficult to quantify. However, some attempts have been made to address this issue. The Competing Values Framework (CVF) is a well-known measurement tool, that offers a clear definition and a consistent analytical framework of OC types. Cameron and Quinn (1999) developed their CVF that consists of four OC types (Clan, Adhocracy, Market and Hierarchy). The CVF is a well-designed instrument that is used frequently and proved to be reliable and valid in OC literature (Fong and Kwok, 2009).

Cameron and Quinn (2011) defined the Competing Values Framework (CVF) as a two-dimensional area that reflects distinct cultural types. First, the flexibility and discretion versus the stability and control axis indicate if the organization focuses on stability or change. The second-dimension deals with whether the organization is externally or internally orientated. Based on these two dimensions, CFV distinguishes four basic cultural types: Clan, Adhocracy, Hierarchy and Market, each culture type’s traits below.

#### Clan Culture

This culture is prevalent in organizations characterized by teamwork and empowering people (Cameron and Quinn, 2011). In this tribal, group, and family culture, the focus is on making flexible internal organizations by engaging committed and loyal employees (Allameh et al., 2011). Furthermore, cohesion and employees satisfaction is more important within these organizations than market and financial objectives (Leal-Rodríguez et al., 2016).

#### Adhocracy Culture

This culture focuses on building up the organization’s ability to respond to environmental changes. The organization is externally oriented with a high level of flexibility. The main feature of this culture is the spirit of entrepreneurship and developing new products and services by ensuring unique resources (Cameron and Quinn, 2011). In this “open system model,” organizations are maneuvering successfully under ambiguity and uncertainty (Machado and Davim, 2019).

#### Market Culture

Organizations with market culture pursue stability and, at the same time, focus on external environment factors like customers, regulators and suppliers to increase productivity and profitability (Cameron and Quinn, 2011). Moreover, in this logical culture, an organization as a market seeks through openness and external focus to make various transactions to achieve a competitive edge and productivity (Allameh et al., 2011; Gomezelj et al., 2011).

#### Hierarchy Culture

This culture type is characterized by concentration, formal rules and the features of the bureaucratic organization. The procedures and policies are well-coordinated and governed (Cameron and Quinn, 2011). Hierarchy culture or “internal process model” is establishing internal stability and organization control, while the business environment is generally stable and anticipated (Machado and Davim, 2019).

### Knowledge Management Processes

Knowledge management (KM) is an essential tool in today’s organizations. There is no unanimous definition in the ongoing debate since KM is a complex and multidisciplinary concept. According to the process perspective, KM is defined and measured by its processes (Alavi and Leidner, 2001). KM is seen as an integrated approach, a complex and loop process to facilitate knowledge-creating, capturing, distributing, storing and using (Cepeda-Carrion et al., 2017; Dalkir, 2017). Much of the research has focused on defining the main processes of knowledge management. Jacks et al. (2012) classifies the knowledge management cycle into four distinct processes: knowledge creation, knowledge storage, knowledge dissemination, knowledge application.

### Organizational Culture Types and Knowledge Management Processes

Researchers widely agreed on the importance of cultural factors in the successful application of KM. Hence, OC is considered as the preeminent complication in KM adoption (Akhavan et al., 2014; Abdi et al., 2018). De Long and Fahey (2000) believed that 80 percent of KM success is about cultural factors. OC mainly defines how organization employees create, share and use their knowledge (Chang and Lin, 2015; Abualoush et al., 2018). According to De Long and Fahey (2000), OC determines which knowledge is essential and why. It also establishes a social interaction context and shapes the way organizational knowledge is created.

#### Organizational Culture Types and Knowledge Creation

Knowledge creation is a good primary answer to why firms exist (Zaim et al., 2019). This process refers to an organization’s ability to update or develop new content, to find better ways of doing tasks more effectively, and to create new knowledge through external sources (Alavi and Leidner, 2001; Allameh et al., 2011). Thus, organizations that have developed effective innovation mechanisms will have the best chance of surviving in turbulent environments.

As illustrated above, adhocracy culture is characterized by a creative workplace and a spirit of taking risks as key values. Therefore, this culture supports creating knowledge in the organization. Otherwise, Hierarchy as an antithesis of Adhocracy is less likely to exist in creative firms. Kayworth and Leidner (2004) argued that organizations with more control and stability often would have difficulties in creating knowledge rather than those seeking to be more flexible and ready to change. While the literature provides convincing arguments about the relationship between Adhocracy, Hierarchy and creation, the effect of a clan or market culture is still largely arguable (Naranjo-Valencia et al., 2016). As such, Mardiana and Tjakratmadja (2016) argued that, in some cases, KM seeks market culture which is suited to encouraging activities that require innovation, since market culture is externally oriented, and external knowledge is critical for innovation. Thus, this typology provides a fertile environment for new ideas (Jantunen, 2005). However, Naranjo-Valencia et al. (2016) stated that market culture also emphasizes stability and control, which may prevent knowledge creation. Furthermore, Clan culture focuses on teamwork attributes, and therefore, it may promote the capability of knowledge creation through collaboration and trust values (Alavi et al., 2005).

**FIGURE 1:**
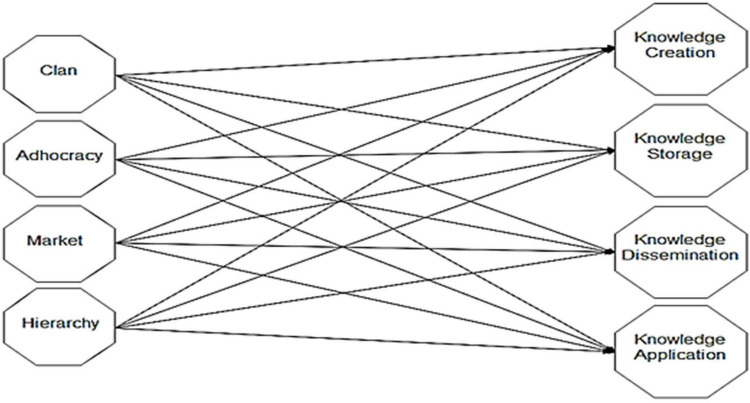
Theoretical framework.

In general, we expect that all culture types except Hierarchy will positively affect knowledge creation. We predict that the effect of market culture will be lower than the effect of Adhocracy and Clan. As a result, the hypotheses listed below were formulated according to the theoretical framework shown in Figure 1.

*H1* _*a*_ *:*
*Adhocracy, Clan and Market cultures have a positive effect on knowledge creation, whereas the hierarchy culture has a negative or weak effect.*

*H1* _*b*_ *:*
*Adhocracy and Clan will have a stronger positive relationship with knowledge creation than market or hierarchy culture.*

#### Organizational Culture Types and Knowledge Dissemination

Knowledge dissemination or sharing, as some authors use these terms interchangeably (Lee V. H. et al., 2016). It refers to the social interaction between employees of an organization, which involves the exchange of individuals’ experiences, implicit and explicit knowledge, thoughts, and suggestions (Castaneda and Ramírez, 2021).

Andam (2017) found no significant impact between adhocracy, hierarchy cultures and knowledge sharing, while clan and market cultures are positively correlated with embedded knowledge sharing in the public sector. Considering that organizations with a hierarchy culture tended to be a “closed system,” Chang and Lin (2015) concluded that this culture type does not support knowledge transfer because people are reticent and not well-motivated. Consequently, the organizational knowledge flow will be prevented. Tseng (2010) suggested that Hierarchy is incompatible with socialization and internalization and is an inconvenient selection for knowledge management strategy, but it is suitable with combination and externalization. Moreover, Sensuse et al. (2015) argued that hierarchy culture obliges employees through formal procedures to disseminate knowledge, whereas clan culture as a friendly workplace is more appropriate for knowledge sharing. Alavi et al. (2005) concur that an organization with a value of collaboration and trust will improve employee readiness to share expertise, skills, and knowledge. In Market culture, where competitiveness and productivity are the central values, there are no specific obstacles to knowledge sharing. However, this culture shapes a rigid framework for determined and contractually rewarded forms of knowledge sharing (Hendriks, 2004).

Generally, we expect that all culture types will positively affect knowledge dissemination and sharing, but with varying degrees. In this line, the relationships between culture types and knowledge dissemination are hypothesized as follows:

*H2* _*a*_ *:*
*Adhocracy, Clan and Market cultures will positively affect knowledge dissemination, whereas the hierarchy culture will have a negative or weak effect.*

*H2* _*b*_ *:*
*Adhocracy and Clan will have a stronger positive relationship with knowledge dissemination than will a market or a hierarchy culture.*

#### Organizational Culture Types and Knowledge Storage

Knowledge storage is a process that includes many aspects, such as archiving, classification, organizing and indexing knowledge to promote access and exploitation for decision making (Akhavan et al., 2014; Abualoush et al., 2018). In addition, storing is necessary to provide the requisite knowledge in time by enhancing organizational memory and knowledge repositoriesı(Abualoush et al., 2018).

Kayworth and Leidner (2004) argued that organizations with more control and stability created knowledge that could be embedded in their daily routines, thus enhancing their storage capabilities. On the contrary, organizations that seek flexibility and agility culture will have more obstacles in embedding knowledge because of the necessity of constant internal adjustments due to changing environments. Accordingly, it is expected that all types of culture affect the storage of knowledge, but in varying proportions, where the impact is more the more toward stability. The we can suggest the following hypotheses:

*H3* _*a*_ *:*
*All organizational culture types (Clan, Adhocracy, Market, Hierarchy) will positively affect Knowledge Storage.*

*H3* _*b*_ *:*
*Knowledge Storage will have a greater positive relationship with Market and Hierarchy than with Adhocracy and Clan.*

#### Organizational Culture Types and Knowledge Application

Knowledge created within an organization is useless until finding the appropriate ways to use it in different activities and problem-solving situations (Ahmady et al., 2016; Abualoush et al., 2018; Alam, 2019). Kayworth and Leidner (2004) noticed that less attention was paid to this process than knowledge sharing because they are closely relevant processes. Thus, similar organizational cultures which support knowledge sharing will logically promote knowledge application (Chang and Lin, 2015). In addition, Kayworth and Leidner (2004) stated that firms with open cultures would have a greater proclivity to apply knowledge among their members than those with closed ones. Likewise, Chang and Lin (2015) argued that the close system as one of hierarchy culture traits does not help organizations use their knowledge effectively.

In sum, it is expected that all culture types will positively affect knowledge application, but with a weak positive effect of hierarchy culture. The relationships between culture types and knowledge application were hypothesized as follows:

*H4* _*a*_ *:*
*All organizational culture types (Clan, Adhocracy, Market, Hierarchy) will have a positive effect on Knowledge Application.*

*H4* _*b*_ *:*
*Adhocracy, Clan, and market cultures will have stronger positive relationships with Knowledge Application than hierarchy culture.*

### The Suitable Culture Type(s) for Knowledge Management Practices

Cameron and Quinn (2011) stated that over time, organizations create a dominant organizational culture as they adapt and respond to environmental challenges and changes. However, some scholars argued that this scenario is rather an exception. Rai (2011) claimed that it is unlikely to find an organization characterized by only one culture type; in order to be effective, it may need to meet all four sets of characteristics to be balanced and perform well. Moreover, Hartnell et al. (2011) suggested that identifying “a dominant” culture type may be of limited utility because CVF’s four culture types do not fully account for organizational culture’s bandwidth. In addition, the management style reflects the cultural type and vice versa. Pasher and Ronen (2011) argued that any “optimal management style” and accordingly “culture type” had to be customized according to each situation, considering that in some cases, organizations need to pursue more internal control or reduce granted autonomy to employees. Thus, it is quite important to explore which culture types are appropriate for KM processes to understand how to establish the right culture to effectively manage knowledge and enhance competitive superiority (Greenwood et al., 2011).

Studies that have examined the relationship between culture types and KM processes have revealed some inconclusive results, especially for Market and Hierarchy (stability and control dimension). For example, hierarchy culture could be positively correlated with KM processes (Devi et al., 2007; Allameh et al., 2011) or negatively correlated (Biloslavo and Prevodnik, 2012; Chidambaranathan and Swarooprani, 2017). Similarly, market culture could have a negative association with KM processes (Biloslavo and Prevodnik, 2012) or a positive association (Cheng and Liu, 2008; Mubin and Latief, 2019).

On the other hand, there is a growing consensus regarding the impact of clan and adhocracy cultures. According to the CVF model, these two cultures foster flexibility and discretion; hence, they are most likely to support KM. Keskin et al. (2005) concluded that clan culture has approximately the same positive effect as the adhocracy culture on tacit oriented KM strategy. Moreover, Jacks et al. (2012) claimed that trust and openness are the most important cultural traits that influenced KM effectiveness, especially knowledge sharing. Thus, adhocracy culture is more likely suitable for KM practices (Greenwood et al., 2011). Finally, Mardiana and Tjakratmadja (2016) based on a qualitative approach and scoring of 16 studies, found that clan culture is typically more appropriate for KM. Their findings remain unaffected even after including the results of Chidambaranathan and Swarooprani (2017) and Mubin and Latief (2019).

As a result, we propose that Clan and Adhocracy will be more suitable for knowledge management.

*H5. Clan and Adhocracy will be more suitable for knowledge management practices.*

## Materials and Methods

As research on a specific topic grows, researchers become more interested in adopting meta-analysis (Bergh et al., 2016). Although meta-analysis has gained wide acceptance in management sciences, organizational behavior research and other related domains (Geyskens et al., 2009; Rosopa and Kim, 2017), its application in these fields is still limited compared to other research areas like clinical science (Sartal et al., 2021). Meta-analysis is defined as the statistical analysis of previous studies’ results on a particular topic; it is used to statistically aggregate all results to achieve more conclusive results than a single study could provide (Card, 2015; Rosopa and Kim, 2017). This statistical approach provides the possibility of obtaining information and statistics that help to generalize the results or to explore new research trends.

In addition, structural equation modeling is well known as a powerful statistical technique to estimate theoretical models. However, it is acknowledged that results from one study are usually insufficient to demonstrate a subject of interest (Cheung, 2021). Consequently, Meta-analytic structural equation modeling (MASEM), resulting from the combination of meta-analysis (MA) and structural equation modeling (SEM), provides a unique and powerful statistical tool to test a set of relationships (not just a single effect size) in the overall model and to assess its goodness of fit using a larger sample size (Bergh et al., 2016).

### Data Collection

Data collection is a critical step in constructing the pooled correlations. First, we must determine the sample of prior studies that investigated the relationships between the study variables: OC and KM processes. In our study, we conducted three meta-analyses to complete the necessary meta-analytic pooled correlation for path analysis. The literature is reviewed to identify the available meta-analyses results that could be used. When we found more than one study, we used the last published study with the largest number of investigated studies. So, we used the pooled correlation matrix in Hartnell et al. (2019) study concerning the relationships among culture types. Then, we carried out the two other meta-analyses. The results of Hartnell et al. (2019) are illustrated in Table 1.

**TABLE 1:** List of meta-analyses in the study.

Study relationships		Source	Meta-analytic correlation matrix ^–^r/ρĈPSTABLEENTER k/N			
Culture types	Already conducted	Hartnell et al., 2019		Clan	Adhocracy	Market
			Clan	–		
			Adhocracy	0.49/0.59 67/10,551	–	
			Market	0.38/0.45 73/11,336	0.50/0.61 66/10,393	–
			Hierarchy	0.39/0.49 60/10,839	0.27/0.35 59/10,346	0.41/0.52 61/11,107
KM processes	To be conducted			
OC types & KM processes	To be conducted			

*r = sample size weighted mean correlation; k = number of studies; N = total simple size.*

To carry out the remaining meta-analyses, we conducted a search of numerous electronic databases, namely SpringerLink, Emerald, ProQuest, Science Direct, JSTOR, EBSCO and Google Scholar, using the appropriate keywords. Concerning the meta-analysis of the relationship between OC and KM, only the studies used the same organizational culture measurement (Clan, Adhocracy, Market, and Hierarchy), and the main knowledge management processes (Knowledge creation, Knowledge storage, Knowledge dissemination, Knowledge application) were selected. Thus, the used keywords are: “corporate culture,” “organizational culture,” “culture,” “organizational climate,” “Competing Values Framework,” “Knowledge management,” “Knowledge management processes.” So, only studies that reported the full correlation matrix and were written in English and published in peer-reviewed journals were chosen. Mainly, we extracted the paired correlation coefficients (r) among KM processes from the following studied (Fugate et al., 2009; Jiang and Li, 2009; López et al., 2009; Andreeva and Kianto, 2011; Gomezelj et al., 2011; Zwain et al., 2012; Fattahiyan et al., 2013; Ho et al., 2014; Ooi, 2014; Donate and Sánchez de Pablo, 2015; Hegazy and Ghorab, 2015; Tan and Chang, 2015; Wahba, 2015; Costa and Monteiro, 2016; Lee V. H. et al., 2016; Martelo-Landroguez and Cepeda-Carrión, 2016; Shaikh and Aktharsha, 2016; Tseng, 2016; Byukusenge and Munene, 2017; Chidambaranathan and Swarooprani, 2017; Ode et al., 2017; Tongsamsi and Tongsamsi, 2017; Vangala et al., 2017; Abualoush et al., 2018; Alyoubi et al., 2018; Ngoc-Tan and Gregar, 2018; Abbas and Sağsan, 2019; Al Ahbabi et al., 2019; Mubin and Latief, 2019; Ode and Ayavoo, 2019; Zaim et al., 2019; Al-Emran et al., 2020). The identified articles were 32 in total, with 6,835 as a sample size. The last extracted bivariate correlations are between KM processes and OC types; relatively few articles were identified. We used the following studies (Cheng and Liu, 2008; Stock et al., 2010; Allameh et al., 2011; Gomezelj et al., 2011; Biloslavo and Prevodnik, 2012; Chidambaranathan and Swarooprani, 2017; Mubin and Latief, 2019). In total, seven articles reported 16 different effect sizes, with 865 as a sample size.

### Data Analysis

To conduct MASEM we adopted the univariate r approach, also known as two-stage correlation-based MASEM, which is popular in management studies and easy to use. Firstly, we constructed the pooled correlation matrix from primary studies by conducting several meta-analyses. This matrix is treated as a covariance matrix, which will be used in the second stage to fit the structural equation model by a standard SEM statistical package such as Amos, Mplus and Stata (Jak and Cheung, 2020; Cheung, 2021).

There are two different models in MASEM. In the fixed-effect model, all studies share a true identical effect size. Conversely, in the random effect model, it is assumed that every included study may have its specific or different effect size due to differences in measurement methods, sampling and other methodological aspects (Borenstein et al., 2010; Cheung, 2015). Some studies suggested that choosing a random effect model is a way to deal with the heterogeneity problem. However, Borenstein et al. (2010) stated that the test of heterogeneity is not a significant indicator for choosing between the two models. Therefore, in this study, we adopted a random effect analysis since it is the best initial selection for conducting meta-analyses (Hartnell et al., 2011) and there is an intention of generalizing the results (Tufanaru et al., 2015).

Comprehensive Meta-Analysis software (CMA) V_2.2_ was used to insert each paper’s r and sample size and convert all *r* values to Fisher *z* values. After conducting all meta-analyses, the average correlation coefficients of every two variables were inserted into a pooled matrix (Table 2), then submitted into STATA to test the hypothesized model using structural equation modeling.

**TABLE 2:** Meta-analytic results of the relationship between knowledge management processes.

Relationships	*k*	*N*	^–^r	CI_95_ LL UL		*z* value	*p* value
Creation → Dissemination	32	6835	0.590	0.532	0.642	15.644	0.000
Creation → Storage	29	6510	0.494	0.424	0.559	11.887	0.000
Creation → Application	31	6849	0.589	0.518	0.652	12.937	0.000
Dissemination → Storage	27	6096	0.568	0.517	0.616	17.414	0.000
Storage → Application	25	5934	0.561	0.467	0.642	9.706	0.000
Clan- → Creation	7	865	0.439	0.173	0.646	3.111	0.002
Clan- → Dissemination	7	865	0.368	0.058	0.614	2.307	0.021
Clan- → Storage	6	494	0.298	0.023	0.531	2.121	0.034
Clan- → Application	7	865	0.358	0.102	0.570	2.698	0.007
Adhocracy → Creation	7	865	0.391	0.140	0.595	2.979	0.003
Adhocracy → Dissemination	7	865	0.392	0.111	0.616	2.678	0.007
Adhocracy → Storage	6	494	0.316	0.233	0.394	7.128	0.000
Adhocracy → Application	7	865	0.361	0.168	0.527	3.551	0.000
Market → Creation	7	865	0.349	0.123	0.540	2.972	0.003
Market → Dissemination	7	865	0.395	0.068	0.645	2.341	0.019
Market → Storage	6	494	0.330	0.078	0.542	2.541	0.011
Market → Application	7	865	0.321	0.121	0.495	3.093	0.002
Hierarchy → Creation	7	865	0.254	0.190	0.316	7.550	0.000
Hierarchy → Dissemination	7	865	0.295	0.233	0.356	8.841	0.000
Hierarchy → Storage	6	494	0.249	0.050	0.429	2.436	0.015
Hierarchy → Application	7	865	0.294	0.231	0.354	8.801	0.000

*k, number of studies; N, the total number of participants; CI_95_, 95% confidence intervals; LL, lower limit of CI95; UL, upper limit of CI95. ^–^r sample size weighted mean correlation.*

## Results

### Meta-Analytic Results

Table 2 presents the results of the overall meta-analysis of bivariate correlations between study variables. It can be noticed that all knowledge management processes are moderately correlated. Also, all correlation coefficients are significant because all 95% confidence intervals exclude zero.

Table 3 summarizes the final pooled correlation matrix, which is arranged from three meta-analyses. This matrix is necessary for conducting path analysis. Average correlations between all variables are positive and range from 0.249 (between hierarchy and knowledge storage) to 0.590 (between knowledge creation and dissemination), showing a range of small to large effects according to Cohen (1988).

**TABLE 3:** Pooled correlation matrix used in path analyses.

	CREAT	STOR	DISS	APPLI	Clan	Adhocracy	Mark	Hiera
CREAT	1							
STOR	0.494	1						
DISS	0.590	0.568	1					
APPLI	0.589	0.561	0.618	1				
Clan	0.439	0.298	0.368	0.358	1			
Adhocracy	0.391	0.316	0.392	0.361	0.490	1		
Market	0.349	0.330	0.395	0.321	0.380	0.500	1	
Hierarchical	0.254	0.249	0.295	0.294	0.390	0.270	0.410	1

### Path Model Results

**FIGURE 2:**
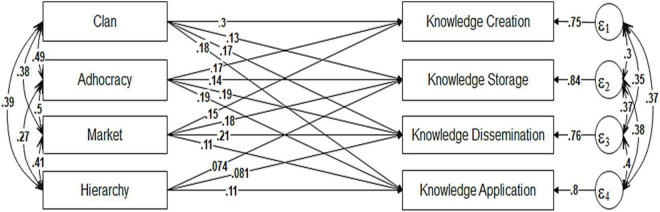
Path analysis results.

Path analysis shown in Figure 2 was conducted to evaluate the overall model and estimate the relationships between the study variables using Stata v.15. First, we have to define the sample size used in addition to the pooled correlation matrix. The harmonic mean in these cases is usually used. According to Bergh et al. (2016), harmonic mean is preferred compared to other options such as the median and arithmetic mean. The harmonic mean of all sample sizes 1,194 was used in the analyses. Preliminary results of the first model showed inadequate fit indices (RMSEA = 0.409, CFI 0.488, SRMR 0.148). Here, we can choose between two types of modifications, whether adding pathways between knowledge management processes or covariances.

It is broadly agreed that KM processes have a strong interdependency (Biloslavo and Prevodnik, 2012). Thus, it is difficult to establish any specific linear relationship between them. Alavi and Leidner (2001) point out that there is no predominant sequential effect between knowledge processes, implying that there is no identical process with which we should always begin or end. Hence, including new paths from one process to another one will not be adequately justified. From this perspective, we made seven modifications in total. First, we removed the path between Hierarchy and creation since Hierarchy was not a significant predictor of knowledge creation; then, we added six covariances between the residuals of KM processes, as visualized in Figure 2.

The alternative model was tested with the following results (the goodness of fit Chi-Square = 0.000, df = 0, *p*-value = 1.00000 > 0.05 and RMSEA (Root mean squared error of approximation) = 0.027 < 0.08, CFI = 1.000, Comparative fit index TLI = 0.992, Tucker-Lewis index SRMR = 0). Thus, the model tested is fit. Good fit indices indicated that meta-analytic data support well our theoretical model. In addition, covariance coefficients between OC types were positive and statistically significant.

Table 4 summarizes the results of path analysis. All the hypothesized paths were positive and significant (*p*-value < 0.01) except for the effect of Hierarchy on knowledge creation. The direct effects on knowledge creation are 0.30, 0.17, 0.15 for Clan, Adhocracy and Market culture, respectively. Respecting the same ordering of culture types, the direct effects on knowledge dissemination are 0.17, 0.19, 0.21, and 0.08. Also, the direct effects on knowledge storage are 0.13, 0.14, 0.18, and 0.07. Finally, the direct effects on knowledge application are 0.18, 0.19, 0.11, and 0.11. In addition, the values of R-squared were 0.25, 0.16, 0.24, and 0.20 for knowledge creation, storage, dissemination and application, respectively.

**TABLE 4:** Standardized path coefficients for study direct relationships.

Relationships	Estimate	Std.Err	*z*-value	*p*-value
Clan → Creation	0.30	0.029	10.22	0.000
Clan → Dissemination	0.17	0.03	5.53	0.000
Clan → Storage	0.13	0.03	4.13	0.000
Clan → Application	0.18	0.03	5.81	0.000
Adhocracy → Creation	0.17	0.03	5.42	0.000
Adhocracy → Dissemination	0.19	0.03	5.53	0.000
Adhocracy → Storage	0.14	0.03	4.31	0.000
Adhocracy → Application	0.19	0.03	5.77	0.000
Market → Creation	0.15	0.029	5.13	0.000
Market → Dissemination	0.21	0.030	6.69	0.000
Market → Storage	0.18	0.032	5.52	0.000
Market → Application	0.11	0.031	3.62	0.000
Hierarchy → Creation	0.03	0.036	1.36	0.172
Hierarchy → Dissemination	0.08	0.025	6.69	0.000
Hierarchy → Storage	0.07	0.028	2.65	0.008
Hierarchy → Application	0.11	0.025	4.33	0.000

## Discussion

This section combines the study findings in order to answer the theoretical hypotheses outlined in the introduction.

First, and as expected, the relationships between all OC types and knowledge creation are positive and significant except for Hierarchy. As discussed above, Adhocratic organizations have an innovative workplace and readiness for change market culture. Biloslavo and Prevodnik (2012) stated that an organization may find it more convenient and cost-effective to target external knowledge sources for specific reasons. The study’s findings comply well with several prior studies (Chidambaranathan and Swarooprani, 2017; María del Rosario et al., 2017). Similarly, the main model results showed that adhocracy and clan cultures are more associated with knowledge creation. Thus, hypotheses *H1*_*a*_ and *H1*_*b*_ were fully accepted.

Second, the results show that all OC types, except hierarchy culture, positively affect knowledge dissemination. Therefore, hypothesis *H2*_*a*_ was fully confirmed, whereas hypothesis *H2*_*b*_ was partially supported. It has been noticed that clan, adhocracy, and market cultures have approximately the same impact (0.17, 0.19, 0.21) on knowledge dissemination. Although the effects of clan and adhocracy cultures were expected, the effect of market culture was somewhat surprising. One possible explanation could be related to the benefits of its external focus, which may enhance its ability to share knowledge with external parties, this could have indirect effects on internal knowledge dissemination. Furthermore, Oh and Han (2020) concluded that this rational culture emphasizes productivity and goal achievement, enhancing group and feedback learning since it is linked more with explicit knowledge (Mardiana and Tjakratmadja, 2016). This culture’s emphasis on traits like internal stability may also enhance knowledge sharing.

Third, the positive relationships between OC types and knowledge storage as outlined in *H3*_*a*_ are also supported, while hypothesis *H3*_*b*_ is partially confirmed since market culture, unlike Hierarchy, has a stronger effect than Clan or Adhocracy. Market culture has a surprising effect on knowledge sharing, as seen earlier, especially on explicit knowledge.

Fourth, *H4*_*a*_ is confirmed in that all OC types positively impact knowledge application, whereas *H4*_*b*_ is partially accepted because the impact of Adhocracy and Clan is greater than that of Market and Hierarchy. Thus, the flexibility and discretion dimension is more important in terms of knowledge application than stability and control.

Finally, hypothesis (H5), which investigates the appropriate organizational culture type for KM processes, is not fully supported. The model results showed that Clan culture is most closely related to knowledge creation, while Adhocracy culture has a stronger effect on Knowledge application, and market culture has a stronger effect on knowledge dissemination and storage. Therefore, hypothesis H5 is not fully supported. However, hierarchical culture, as expected, has the lowest impact on all KM processes. Thus, this culture hinders KM since it is very formalized and depends on strict procedures. This result aligns with many prior studies such as Devi et al. (2007). However, several studies like Nuñez Ramírez et al. (2016) found that Hierarchy has a stronger effect on KM, as this type of culture can be convenient in some cases. Mardiana and Tjakratmadja (2016) mentioned that Hierarchy would be suitable in difficult organizational conditions such as crisis time. In this sense, Felipe et al. (2017) pointed out that hierarchy culture may produce a temporary success in such time but will prevent the adaptation and innovation ability in the long term.

Even though Clan and Adhocracy are known as suitable culture types for KM practices, the study findings show that no particular culture type among Clan, Adhocracy and Market has more substantial effects on all KM processes. Thus, cultural traits that include both flexibility and discretion or external focus and differentiation are needed in the success of KM application. In this vein, understanding an organization’s culture profile will assist in determining the required improvements or changes (Rostain, 2021). For instance, in the public sector, hierarchy culture is naturally ingrained and hard to be eliminated, then it should be balanced by the other culture types (Devi et al., 2007). Therefore, the success of KM processes is determined by the ability to develop a balanced portfolio of cultural traits throughout the three cultural types. A strict tendency toward a particular culture type within an organization should be avoided as it is expected to affect KM negatively.

Additionally, the positive relationships between all KM processes align with theory and agree well with existing studies. Otherwise, Hartnell et al. (2011) argued that the positive interrelationships between all culture types are not suitable with CVF theory. It is presumed that clan culture will have a negative or insignificant relationship with market culture and, similarly to the association between adhocracy and hierarchy cultures. Hartnell et al. (2019) found the same results in their meta-analytic study. For Hartnell et al. (2011), this may result from the common method bias problem. This case can be further understood and analyzed in the model assessment step. We can investigate using cross-loading matrix (among other ways) how one of the CVF components is related to the others and if analyzing the common method bias problem (many previous studies that adopted SEM do not even report the results of this step) can give a reasonable explanation. Although this is hard to be verified in our study, it is an essential consideration for future studies, through the need to verify the existence of this problem, especially in the phase of measurement model assessment.

## Conclusion, Limitations and Future Research

The purpose of the current study was to demonstrate the effect of OC types on KM processes and add value to the existing literature. The findings indicate that three OC types significantly affect KM processes but with various extents of effect strength. Clan and adhocracy cultures are generally proper for knowledge creation and application. In contrast, market culture is more associated with knowledge dissemination and storage, while Hierarchy is not preferred because of its weak effects. The study findings suggest that there is no single culture type that is entirely suitable for KM practices. These findings may have some important implications in managerial practices. If an organization is aware of the dominant culture’s characteristics and its influence on KM processes, this may cause a better understanding of which cultural traits are needed in order to improve KM processes.

Our findings should be applied only to the specific aspects investigated in this study. The first aspect relates to the limitation of using meta-analysis in general and meta-analytic path analysis in particular. A limitation of any meta-analysis is its dependence on previously published primary research, which may not contain sufficient or complete data (Earnest et al., 2011). Another limitation of the finding is the limited number of included studies. Likewise, the study results are limited by adopting the MASEM approach. For instance, one of the common shortcomings of the univariate r approach is treating the correlation matrix the same as the covariance matrix to test the model (Jak and Cheung, 2018). Consequently, further research could compare the results with MASEM alternative approaches such as generalized least squares (GLS).

The second aspect is regarding the limitations of the study itself. Although numerous studies used CVF to measure the organizational culture, relatively few used this taxonomy in the relationship with KM processes. Thus, future research should consider including more studies, and further empirical research would be more beneficial to confirm the results. In addition, several studies ignored the effect of control variables inclusion. For instance, it is essential to insert the environment characteristics or organization type as control variables when studying a sample of different sector organizations. Another significant control variable is firm age, Gomezelj et al. (2011) stated that typically young organizations tend to adopt market culture since innovation needs to be stressed. Furthermore, the type of organization sector is an important control variable since those numerous studies showed that public organizations usually depend on hierarchy culture, which may justify why hierarchy culture has a negligible effect on KM processes. As a result, future studies should seek to address this issue by using appropriate control variables.

Another point of criticism is that the CVF is unable to categorize all aspects of culture (Hartnell et al., 2019). Future work may examine the impact of cultural change if the organization falls in-between (in the transition phase from one type to another) due to competition or entering new markets. This leads to the importance of studying the relationship between an organization’s life cycle and adopting a particular organizational culture type. As such, this study tried to shed light on the organizational culture types and their relationship with each KM process; it did not investigate why every culture is dominant in an organization in the first place.

In addition, the study’s findings revealed that every organization should be aware of its dominant cultural attributes in order to make meaningful changes to improve necessary KM processes. Nevertheless, cultural change is a challenging task, which any organization cannot undertake. Park et al. (2004) suggested that aligning the KM system with OC may be more effective than striving to change the culture itself. This suggestion needs further investigation if OC and KM have a reciprocal relationship.

## Data Availability Statement

The original contributions presented in the study are included in the article/supplementary material, further inquiries can be directed to the corresponding author.

## Author Contributions

All authors listed have made a substantial, direct, and intellectual contribution to the work and approved it for publication.

## Conflict of Interest

The authors declare that the research was conducted in the absence of any commercial or financial relationships that could be construed as a potential conflict of interest.

## Publisher’s Note

All claims expressed in this article are solely those of the authors and do not necessarily represent those of their affiliated organizations, or those of the publisher, the editors and the reviewers. Any product that may be evaluated in this article, or claim that may be made by its manufacturer, is not guaranteed or endorsed by the publisher.
